# A Fourteen-day Experience with Coronavirus Disease 2019 (COVID-19) Induced Acute Respiratory Distress Syndrome (ARDS): An Iranian Treatment Protocol

**DOI:** 10.22037/ijpr.2020.113337.14239

**Published:** 2020

**Authors:** Hamidreza Jamaati, Farzaneh Dastan, Payam Tabarsi, Majid Marjani, Ali Saffaei, Seyed MohammadReza Hashemian

**Affiliations:** a *Chronic Respiratory Diseases Research Center, National Research Institute of Tuberculosis and Lung Diseases (NRITLD), Shahid Beheshti University of Medical Sciences, Tehran, Iran. *; b *Department of Clinical Pharmacy, School of Pharmacy, Shahid Beheshti University of Medical Sciences, Tehran, Iran. *; c *Clinical Tuberculosis and Epidemiology Research Center, National Research Institute of Tuberculosis and Lung Diseases (NRITLD), Masih Daneshvari Hospital, Shahid Beheshti University of Medical Sciences, Tehran, Iran. *; d *Student Research Committee, Department of Clinical Pharmacy, School of Pharmacy, Shahid Beheshti University of Medical Sciences, Tehran, Iran.*

**Keywords:** COVID-19, Coronavirus, Acute respiratory distress syndrome, Treatment

## Abstract

COVID-19 is currently causing concern in the medical community as the virus is spreading around the world. It has a heavy global burden, particularly in low-income countries. The clinical spectrum of COVID-19 pneumonia ranges from mild to critically ill cases and Acute Respiratory Distress Syndrome. An expert panel was held and an internal protocol was developed to manage the COVID-19 induced ARDS according to WHO recommendations and NIH guidelines. Different therapeutic regimens were employed on this protocol based on the ARDS severity and the patients’ special characteristics. The mortality rate, the rate of survivors, and non-survivors were reported. Of the 231 suspected cases of COVID-19 admitted to the hospital during two weeks, 72 patients were admitted to ICU with diagnosis confirmed by RT-PCR. In total, mortality in the ICU was 25% (n = 18) among ARDS patients over two weeks. COVID-19 induced ARDS is a major concern. The rapid progression of ARDS needs specific protocol based on patients’ characteristics and rapid action.

## Introduction

In December 2019, the city of Wuhan in Hubei Province, China, became the center of an outbreak of pneumonia of unknown cause. On January 7, 2020, the Chinese health officials confirmed the identification of a novel coronavirus (COVID-19). COVID-19 from Wuhan in China is currently causing concern in the medical community as the virus is spreading around the world. So far, 102,242 cases of COVID-19 have been clinically confirmed and 3,497 deaths have occurred till March 7, 2020. COVID-19 has a significant burden worldwide as a pandemic disease. The clinical presentation of COVID-19 pneumonia may present from mild to severe illness including Acute Respiratory Distress Syndrome (ARDS). Remarkably, the critically ill patients with COVID-19 are more likely to develop ARDS regarding the cytokine cascade activation over a short period of time (1, 2). The most common complication is ARDS, which is seen in the COVID-19 patients with high mortality rates. 

## Experimental

**Figure 1:**
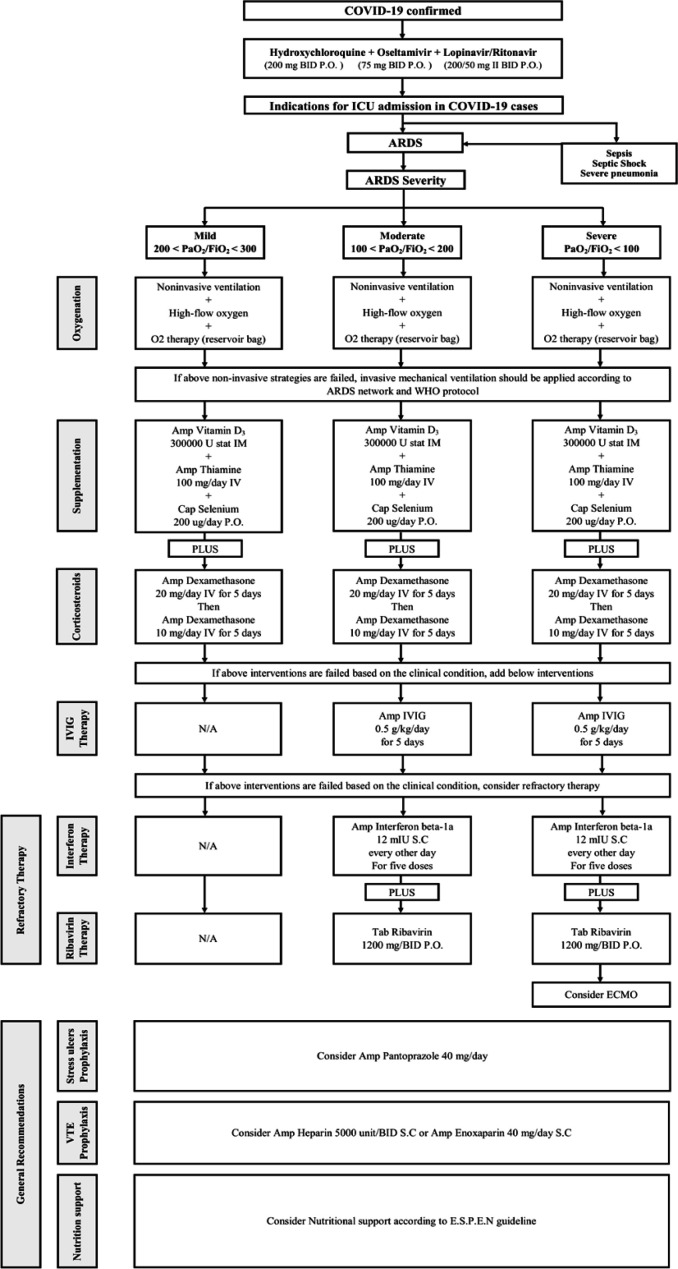
Treatment protocol of COVID-19 induced rapid acute respiratory distress syndrome. ARDS: Acute Respiratory Distress Syndrome; ECMO: Extracorporeal Membrane Oxygenation; IVIG: Intravenous immunoglobulin; VTE: Venous Thromboembolism; WHO: World Health Organization

On February 19, 2020, Iran reported its first confirmed cases of COVID-19 and Dr. Masih Daneshvari Hospital was selected as a referral center for COVID-19 cases. The hospital was equipped with a special set-up to admit COVID-19 cases. On February 20, 2020, seven patients were admitted to the hospital with the confirmed COVID-19. In the first 30 h, four cases COVID-19 who were referred from the other hospitals and admitted in the intensive care unit (ICU) died due to severe ARDS. Because of the rapid progression of ARDS, an expert panel was held on 22 February 2020, consisting of all intensivists, infectious disease specialists, pulmonologists, internal medicine specialist, cardiology specialist, and clinical pharmacy specialist. Finally, an internal protocol was developed to manage the COVID-19 induced ARDS according to WHO recommendations and NIH guidelines (3). Different therapeutic regimens were employed on this protocol based on the ARDS severity and the patients’ special characteristics. This protocol is shown in Figure 1.

Of the 231 suspected cases of COVID-19 admitted to the hospital during two weeks, 72 patients were admitted to ICU with diagnosis confirmed by RT-PCR. In total, mortality in the ICU was 25% (n = 18) among ARDS patients over two weeks (Table 1).

**Figure 2:**
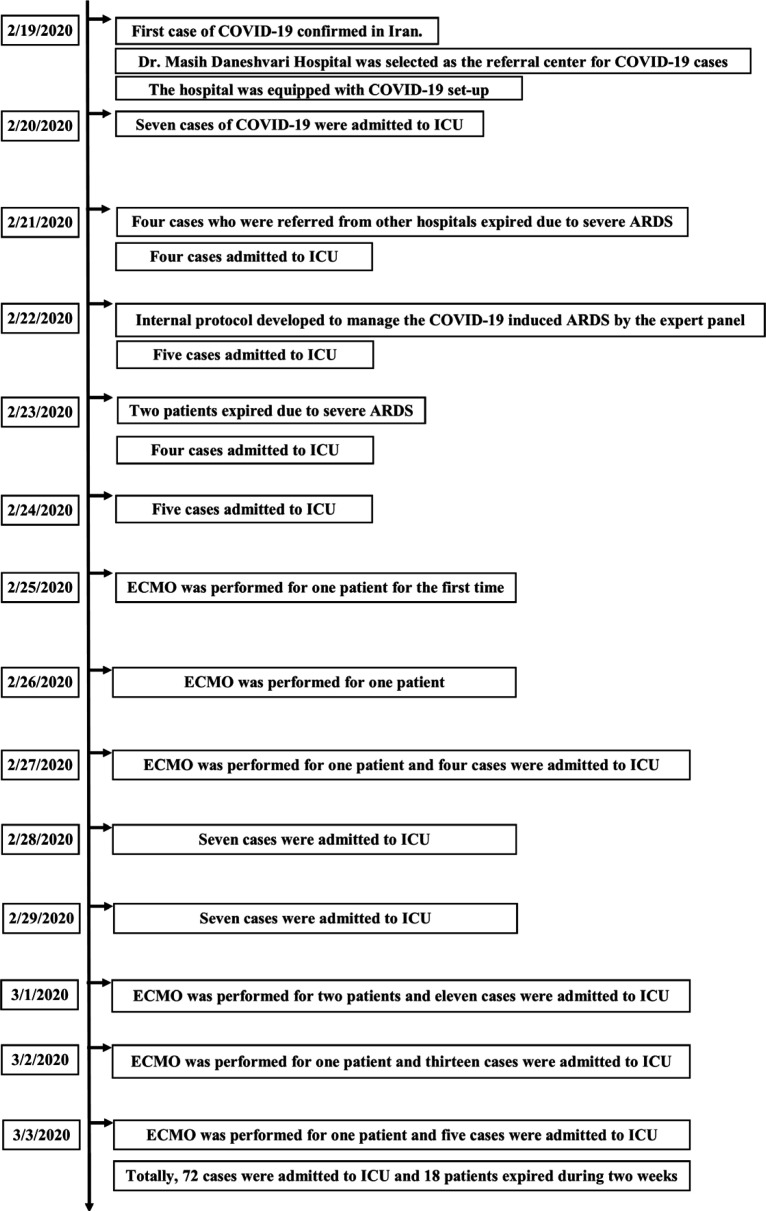
The timeline of two-week events in Dr. Masih Daneshvari Hospital

Twenty-four patients recovered and were transferred to the ward. Thirty patients were under treatment in ICU. Totally, 29 patients expired during two weeks including 18 cases in ICU. The timeline of two-week events is depicted in Figure 2. 

**Table1:** Fourteen days mortality at a glance

Non-Survivor (%)	Survivor (%)	Number (%)	Group
3 (16.7)	18 (33.3)	21 (29.2)	Mild ARDS
4 (22.2)	31 (57.4)	35 (48.6)	Moderate ARDS
11 (61.1)	5 (9.3)	16 (22.2)	Severe ARDS
18 (100)	54 (100)	72 (100)	Total

ARDS: Acute Respiratory Distress Syndrome.

## Results and Discussion

*Protocol Recommendations*

The patients’ vital signs, CBC, serum creatinine, urea, urine output, CRP, LFT, bilirubin, coagulation parameters, ABG, and regular chest imaging must be closely monitored.

All of the patients were administered hydroxychloroquine, oseltamivir, and lopinavir-ritonavir before admission to ICU based on the Ministry of Health protocol and recent data regarding COVID-19 treatment (4, 5).

*Oxygenation*

Oxygen therapy, non-invasive ventilation or invasive mechanical ventilation are considered based on O2 saturation according to the WHO recommendations and NIH guidelines. The patients were categorized into three groups based on PiO_2_/FiO_2_. According to Berlin criteria, mild, moderate, and severe ADRS are defined as PiO_2_/FiO_2_ between 200 and 300, 100 and 200, and less than 100, respectively. Invasive mechanical ventilation should be applied to ARDS patients with persistent hypoxemia. 

*Supplementation*

We recommend the administration of vitamin D3, thiamine, and selenium in the ARDS patients according to our protocol. Vitamin D deficiency is associated with greater cellular inflammation and cytokine release at 48 h after ARDS development. Thiamine deficiency is also a risk factor for ARDS development based on several published trials, whereas selenium has a beneficial role in oxidative stress pathways.

*Steroid therapy*

As we have faced with the rapid progression of ARDS in our cases, the administration of corticosteroid is recommended for all our patients to inhibit the inflammation process in ARDS. High doses of dexamethasone as 20 mg daily from day 1 to 5, then 10 mg daily from day 6 to 10 were administered in all stages of ARDS, even in the mild cases. This approach was applied based on the study done by Villar *et al.* and the rapid progression of ARDS in our cases in order not to lose the time (6).

*Immunoglobulin Therapy*

IVIG is administered in the cases that failed with the above strategies. IVIG has controversial effects on the patients with respiratory coronavirus. IVIG is applied to treat some diseases, especially primary immune deficiencies, autoimmune neuromuscular disorders, and respiratory failure regarding sepsis. It is considered as an effective agent in the cases of coronavirus associated fulminant myocarditis (7, 8).

*Interferon beta-1a and Ribavirin Combination*

Combination therapy with interferon Beta-1a and ribavirin in all refractory cases is administered based on data regarding coronavirus treatment and Ebola experiences.

*Extracorporeal membrane oxygenation (ECMO) and Refractory Therapy*

ECMO is a specialized, resource-intensive, and expensive form of life support. However, it is associated with severe complications including nosocomial infection and hemorrhage. ECMO can serve as a life-saving rescue therapy for refractory respiratory failure in the setting of ARDS, such as that induced by coronavirus disease 2019 (COVID-19) (9). 

*Other therapies*

Stress ulcer prophylaxis, venous thromboembolism prophylaxis, and nutrition management are applied to all of the patients.
